# The association between statin use and risk of age-related macular degeneration

**DOI:** 10.1038/srep18280

**Published:** 2015-12-14

**Authors:** Le Ma, Yafeng Wang, Junhui Du, Mingxu Wang, Rui Zhang, Yihao Fu

**Affiliations:** 1 School of Public Health, Xi’an Jiaotong University Health Science Center, Xi’an, China; 2 Key Laboratory of Environment and Genes Related to Diseases (Xi’an Jiaotong University), Ministry of Education of China, Xi’an, China; 3 Xi’an Ninth Hospital, Xi’an Jiaotong University Health Science Center, Xi’an, China; 4 Australian School of Business, University of New South Wales, Sydney, Australia

## Abstract

The aim of the present study was to evaluate the association between statin use and the risk of age-related macular degeneration (AMD). A systematic search of the PubMed, EMBASE and ISI web of science databases was used to identify eligible published literatures without language restrictions up to April 2015. Summary relative ratios (RRs) and 95% CIs were estimated using a fixed-effect or random-effects model. A total of 14 studies met the inclusion criteria and were included in this meta-analysis. No significant association was observed between statin use and the risk of any AMD (RR, 0.95; 95% CI, 0.74–1.15); and stratified analysis showed that statins had a significantly different effects on early and late stages of AMD. For early AMD, statin use significantly reduced the risk approximately 17% (RR, 0.83; 95% CI, 0.66–0.99). At the late stage, we observed a significant protective association of statin use with exudative AMD (RR, 0.90; 95% CI, 0.80–0.99), in contrast with the absent association between statins and geographic atrophy (RR, 1.16; 95% CI, 0.77–1.56). These results demonstrated that statin use was protective for early and exudative AMD. Additional large prospective cohort studies and RCTs are required to determine the potential effect of statins on AMD prevention.

Age-related macular degeneration (AMD) is a progressive eye disorder and the leading cause of visual impairment among the individuals over 50 years of age in western countries^1^. The common features of early AMD include the the presence of drusen and pigmentary abnormalities in the retinal pigment epithelium (RPE); and late AMD is manifested through geographic atrophy or the development of neovascularization^2^. Approximately 8.7% of the world population suffers from AMD, and the projected number of people with this disease is approximately 196 million individuals in 2020, increasing to 288 million in 2040^3^, presenting a growing public health problem among the aging population^4^.

Although the pathogenesis of AMD remains elusive, inflammation and oxidative damage have also been implicated as playing a crucial role in this disease^5^. Epidemiological studies have demonstrated that AMD and cardiovascular disease share many risk factors, such as smoking, elevated serum cholesterol, atherosclerosis and hypertension^6^,^7^. Thus, the drugs lowering the risk of cardiovascular disease might also confer a protective effect for AMD. As hydroxymethylglutarylcoenzyme A (HMGCo-A) reductase inhibitors, statins reduce low-density lipoprotein (LDL) levels and exert anti-inflammatory effects, in addition to modifying dyslipidemia, both of which are relevant in the development of AMD, indicating that statin use might play a potential role in reducing the risk of disease^8^,^9^. Recently, many studies have investigated the relationship between statin use and the risk of AMD; however, the results of these studies have been inconsistent^10^,^11^. In addition, the pathological hallmarks of the disease might differ during early and late AMD; and whether the effect of statin use is controversial during different stages remain unclear^12^.

Therefore, we conducted a meta-analysis to pool the results of all available association studies between statin use and the risk of AMD. Furthermore, stratified analyses were also conducted to explore associations with differentiation in AMD subtype.

## Results

**Figure 1:**
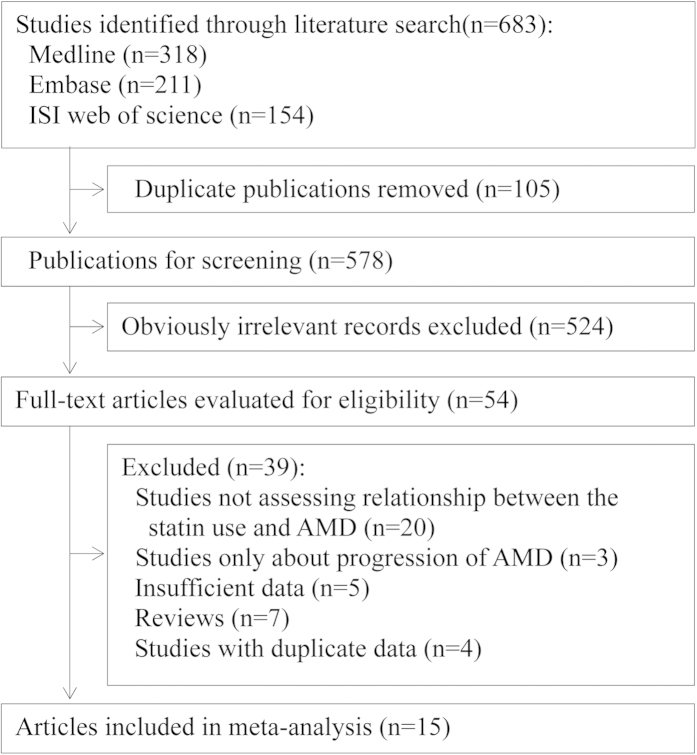
Flowchart for the selection of eligible studies. AMD, age-related macular degeneration.

After de-duplication, the database search yielded 578 records, screened according to the titles and abstracts, with 54 records determined as potentially eligible (Fig. 1). The full texts and data integrity of these studies were reviewed, and 14 articles were included in this meta-analysis^10^,^11^,^13^,^14^,^15^,^16^,^17^,^18^,^19^,^20^,^21^,^22^,^23^,^24^.

### Characteristics of the studies

The characteristics of the included studies are presented in Table 1. Among these studies, seven studies were cohort studies, five studies were case-control studies and three studies were cross-sectional studies. The number of subjects ranged from 744 to 104,176. All studies were conducted in Caucasians, except for one study involving in Asian individuals. The average age of subjects ranged from 52.0 to 80.2 years. The study population in thirteen studies included both men and women, and one study was entirely comprised of men. The diagnosis of AMD was based on fundus photography in thirteen of the studies, and in one study, eye specialists diagnosed AMD. Eleven studies used WARMGS criteria to establish AMD, and the ICGS was also applied in three studies. Most studies controlled for some conventional risk factors, including age (n = 14), smoking (n = 9) and body mass index (n = 6), and three studies adjusted for serum lipids.

### Statin use and AMD

**Figure 2:**
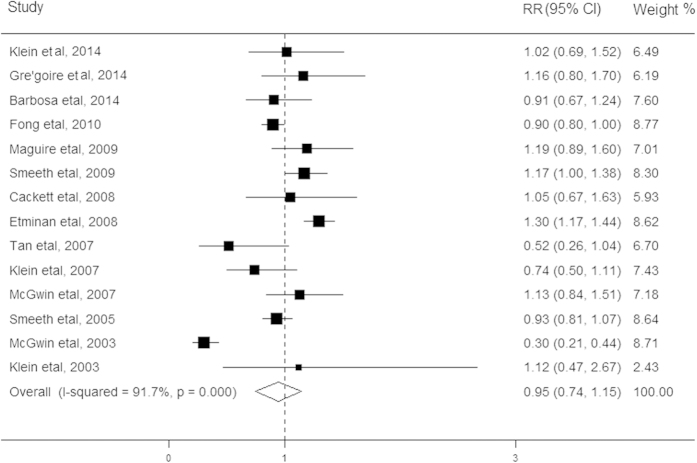
Forest plot on the associations between statin use and any age-related macular degeneration. The boxes and lines indicate the relative risks (RRs) and their 95% confidence intervals (CIs) on a log scale for each study. The pooled relative risk is represented by a diamond. The size of the black squares indicates the relative weight of each estimate.

The relationship between statin use and risk of AMD was evaluated in fifteen studies, comprising 235,021 participants (Table 1). Among these studies, twelve studies showed no association between statin use and AMD, one study showed a significantly reduced risk of this disease while one study showed a significantly increased risk of the AMD. The pooled results revealed that users of stain compared with non-users conferred a summarized RR of 0.95 (95% CI, 0.74–1.15; Fig. 2), using the random effect model. Significant heterogeneity was detected across these studies (I^2^ = 91.7%; *P* < 0.001). When excluding cross-sectional studies in the secondary analysis, this association was not appreciably altered (RR, 0.94; 95% CI, 0.72–1.16). The results of stratified analyses showed significant inconsistency in the direction of effect when studies were grouped according to early and late stages and the I^2^ for statistics decreased from 91.7% to 0%. However, the other characteristics of participants shared consistency in the direction of effect. In addition, the pooled RRs remained stable after removing each study one at a time. We did not observe any evidence for the presence of publication bias in the eligible studies (Begg’s test *P* = 0.57; Egger’s test *P* = 0.20).

### Statin use and early AMD

**Figure 3:**
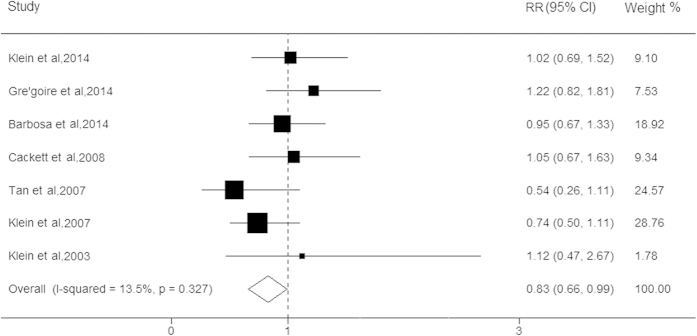
Forest plot on the associations between statin use and early age-related macular degeneration. The boxes and lines indicate the relative risks (RRs) and their 95% confidence intervals (CIs) on a log scale for each study. The pooled relative risk is represented by a diamond. The size of the black squares indicates the relative weight of each estimate.

Seven studies reported data on the association between stain use and early AMD, with a total of 37,308 participants. Although most of these studies showed a protective tendency, none of these studies showed any significant association between statin use and early AMD. When the results of these studies were pooled into the meta-analysis, the use of statins was significantly associated with lower risk for early AMD (RR, 0.83; 95% CI, 0.66–0.99; I^2^ = 0%, *P* = 0.33; Fig. 3). The results of the stratified analysis based on cohort study showed that the association between statin use and early AMD remained robust (RR, 0.77; 95% CI, 0.56–0.97; Table 2). In addition, the stratified analysis according to early AMD subtypes was performed and the results revealed that individuals with statin use had approximately 45% and 16% lower risks of soft indistinct drusen (RR, 0.55; 95% CI, 0.29–0.81) and large drusen (RR, 0.84; 95% CI, 0.68–0.99), respectively. The sensitivity analysis showed that the significant relationships among the pooled RRs remained stable between stain use and early AMD. No evidence for the presence of publication bias was detected (*P* > 0.05).

### Statin use and late AMD

**Figure 4:**
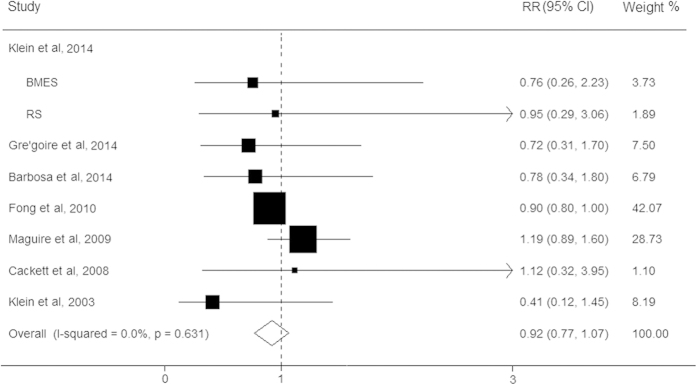
Forest plot on the associations between statin use and late age-related macular degeneration. The boxes and lines indicate the relative risks (RRs) and their 95% confidence intervals (CIs) on a log scale for each study. The pooled relative risk is represented by a diamond. The size of the black squares indicates the relative weight of each estimate. BMES, the blue mountains eye study; RS, the rotterdam study.

We subsequently evaluated the effect of statins on the risk for late AMD in eight studies, with a total of 22,973 participants. The present results showed that no significant associations of statin use with the risk for late stage AMD were observed (RR, 0.92; 95% CI, 0.77–1.07; I^2^ = 0%; *P* = 0.63; Fig. 4). The pooled RR of late AMD based on cohort studies could did not substantially differ (RR, 0.95; 95% CI, 0.68–1.22). However, stratified analyses showed that the use of stains conferred a significant decrease for CNV risk (RR, 0.90; 95% CI, 0.80–0.99), whereas no effect was observed for GA (RR, 1.16; 95% CI, 0.77–1.56). For CNV, the results of stratified analysis based on cohort studies found that statin use had a protective tendency for the risk of this disease, but did not reach statistic significant (RR, 0.82; 95% CI, 0.41–1.23). Moreover, no publication bias was detected (*P* > 0.05).

## Discussion

The results from this meta-analysis showed that statin use was significantly associated with lower risk for early AMD compared with non-users. Moreover, in contrast with the absent association between statins and GA, we observed a significant association of statin use with exudative AMD, indicating that statins might have a protective role in the prevention of early AMD and exudative AMD.

AMD and cardiovascular disease share many common pathophysiological mechanisms, and low-density lipoprotein cholesterol (LDL) has been detected in the retinal pigment epithelium (RPE) and Bruch’s membrane, for which deposition is thought with be analogous to that in the arterial intima in atherosclerosis^25^. The previous study identified that inflammation through the production of mature mediators could initiate inflammasome formation in the RPE, thereby mediating cell death, and ultimately leading to epithelium degeneration^26^. As the first-line drug therapy for the prevention of cardiovascular disease, statins are a class of medications that reduce serum LDL cholesterol^25^,^27^. Increasing laboratory-based studies have demonstrated that statins exert anti-inflammatory, antioxidant, and pleiotropic effects that might reduce the risk of AMD. Recently, multiple studies have been conducted to evaluate the effects of statin use and AMD development or progression; however, the results from the published medical literature relating to the association of statin intake and AMD remained controversial. A previous meta-analysis including seven studies was performed in 2007, which showed no association between statin and the risk of this disease, consistent with previous results^28^; however, the most of these studies were absent of AMD classification and could not elaborate the relationship between statin use and the risk of AMD subtypes. Due to different pathogenic mechanisms, the potential effects of statin intake might differ between early and late AMD^29^. This hypothesis was also supported through the finding of the stratified analysis showing that statin use had a more protective effect during early AMD compared with late stage AMD. In addition, inconsistent with the source of heterogeneity in the previous meta-analysis entirely ascribed to the study of McGwin *et al.*, there was no evidence of significant heterogeneity in the present study after performing a stratified analysis based on AMD subtypes, indicating that the different clinical stages of this disease might be the main source of heterogeneity. Therefore, it is necessary to evaluate the relationships between statin use and different stages of AMD.

In the present study, statin use could significantly reduce the early AMD risk by approximately 13%, primarily contributing to the lipid-lowering effects of this drug. Curcio *et al.* showed that statin-mediated reduction in serum LDL cholesterol levels might decrease LDL cholesterol to deposit in drusen^30^. In addition to lipid-lowering effects, statins also show anti-inflammatory through the suppression of the activation of the inflammatory pathway in macrophages. Furthermore, statins decrease the plasma concentrations of C-reactive protein (CRP) through the reduction of the number of inflammatory cells and inflammatory cell functions^31^,^32^. As an inflammatory marker, higher concentrations of CRP could reduce the deactivation of the complement cascade, resulting in alterations in the RPE and damage to the underlying Bruch’ s membrane; and a cascade of decreased levels and drusen accumulation^33^. This result was consistent with Seddon *et al.*, showing that elevated CRP levels were independent risk factors in the pathogenesis of AMD^34^. Therefore, statin use might play a more prominent role in drusen initiation, which could retard the onset and progression of early stage AMD.

In contrast with the absent effect of statins for GA, the results revealed a significant risk reduction for exudative AMD in statin users, likely reflecting the anti-inflammatory effects of statins. Chronic inflammation could lead to endothelial dysfunction and macrophage activations, and activated macrophages secrete chemokines and vascular endothelial growth factor, causing further cellular damage, Bruch’s membrane degradation and choroidal neovascularization, which have been implicated in the development of this disease^25^,^35^. However, macrophages and other leukocytes might be inefficiently involved in the formation of GA, which is primarily caused by the loss of retinal pigment epithelium and photoreceptors^36^. Statins remit chronic inflammation after relieving the interactions between macrophages, modified lipoproteins, and some elements of the retinal arterial wall, which might partly explain why statin significantly reduces the risk of CNV rather than GA.

Some potential limitations of the present study merit consideration in interpreting the findings. First, although these study designs might be inherently biased through various factors due to the observational nature of the studies included, the combined sample size was relatively large, potentially adding to the strength of this analysis. Second, although the present results showed that statin use could significantly reduce the risk of CNV, the results based on cohort studies found that statin use only had a protective tendency for this AMD subtype, indicating that such association still needed to be investigated to confirm. Third, although these results were based on adjusted estimates, these studies differed in adjusted covariates, and a more precise analysis should be conducted when individual data are available, thereby facilitating adjustment through more potential confounding factors. Forth, the definition of AMD varied between studies and various grading systems have been used in different countries and during different periods in which the studies were performed. Although the RR of AMD associated with statin use did not substantially differ after stratified analysis, the different criteria of this disease might also affect the present results. Finally, the potential publication bias was also a concern. Although we did not observe apparent publication bias through statistical tests, it was still difficult to completely rule out this problem because there was no an abundant amount of studies to adequately detect biases.

In conclusion, the meta-analysis demonstrated a protective association of statin use and early AMD, regardless of soft indistinct drusen or large drusen. Moreover, compared with the absent effect of statins and GA, statin use might reduce the risk of exudative AMD. To date, the association of statin use with subtypes of AMD still required to be confirmed, especially for CNV. Moreover, there is insufficient evidence from RCTs suggesting that statin use might delay the onset of AMD, and the data from RCTs could provide insight into the role for statins in AMD prevention. Therefore, further well-designed large prospective cohort studies and RCTs are warranted before definitive conclusions can be drawn regarding the potential effects of statins on AMD prevention.

## Methods

### Search strategy

A computerized literature search of MEDLINE, EMBASE, and ISI web of science databases through April 2015 was conducted to identify all relevant articles involving the association of stain use with AMD, using the search terms: (“antilipidemic agents” or “anticholesteremic agents” or “hydroxymethylglutaryl-CoA reductase inhibitors” or “statin”) and (“AMD” or “age-related maculopathy” or “neovascular AMD” or “exudative AMD” or “choroidal neovascularization (CNV)” or “geographic atrophy (GA)” or “macular degeneration”). No language restriction was applied for searching and study inclusion, and we assessed the reference lists of retrieved articles and relevant reviews for additional published and unpublished data. When datasets were incomplete for the required data, corresponding authors were contacted for additional information.

### Study selection

Articles that met all of the following criteria were included: 1) cohort studies, case-control or cross-sectional were published as an original article; although randomized clinical trials (RCTs) would have been considered, no trials were available; 2) the study objective was to evaluate the relationship between stain use and AMD; 3) the definition of AMD had to be clearly stated; and 4) studies were also required to present the relative risks (RRs) or odds ratios (ORs) or hazard ratios (HRs) with their 95% confidence interval (CI) or sufficient data to calculate these. When multiple publications reported on the same or overlapping data, we only included the publication with the most subjects. According to the sample size and different stages of AMD, the BMES by Tan *et al.* (2007) was included to investigate the association of statin use with early AMD, whereas the BMES by Klein *et al.* (2014) was selected for inclusion to assess the association of statin use with overall AMD and late AMD (GA and CNV). Additionally, if the studies performed the analysis with different adjustments, the studies with the maximally adjustment were included for the meta-analysis. Three investigators (Y.-F. W., J.-H. D. and R. Z.) independently assessed the retrieved studies. Any inconsistencies were resolved through consensus with a third author (L. M.) for adjudication.

### Data extraction and quality assessment

We extracted the following information from the eligible studies: first author, year of publication, study design, number of subjects, average age, sex distribution, diagnosis method, classification criteria, type of macular degeneration, the RRs (ORs or HRs) overall and in each subgroup and the corresponding CI, and the confounding factors were matched or adjusted in the studies. When a study provided several risk estimates, the most completely adjusted estimate was extracted.

Because no available RCTs were intensified, the Quality of Reporting of Meta-analyses guidelines is not directly appropriate^37^. Therefore, we conformed to the Meta-analysis Of Observational Studies in Epidemiology (MOOSE) guidelines. The studies were categorized as good quality when at least half of the criteria were clearly described and accounted for, as studies that met less than half criteria were considered fair quality or poor quality^38^. Three authors (Y.-F. W., J.-H. D. and R. Z.) independently extracted data and assessed study quality; and any discrepancies were resolved through discussion.

### Outcome measurement

The definition of AMD varied between studies using International Classification and Grading System (ICGS) and Wisconsin Age-related Maculopathy Grading System (WARMGS). Briefly, early AMD (grades 2 and 3) was defined as the appearance of indistinct soft/reticular drusen or large distinct soft drusen (≥125 μm in diameter). Late AMD was defined as presence of either CNV or GA. Any AMD was defined in participants as the appearance at early or late AMD. For studies reporting results separately in early and late stages, the risk estimates were pooled as any AMD prior to the inclusion of the study in the overall analysis.

### Statistical analysis

The measure of effect of interest is the RR with 95% CI which is an intuitive and commonly used measure in the medical and public health literature. Because AMD is not common, ORs, HRs or RRs could be considered an approximation of relative risk. The associations between statin use and the risk of AMD were estimated after calculating pooled RRs and 95% CI, using a fixed effects model or a random effects model according to the heterogeneity. Heterogeneity among individual studies was evaluated by calculating the Cochran’s Q statistic and the I^2^ test (*P* < 0.10 indicates an existence of heterogeneity and I^2^ > 50% indicated a statistical significance). We performed a meta-analysis using all observational studies; and only cohort and case-control studies were identified and included in the secondary analysis to be discussed later. In case of significant heterogeneity, the sources of heterogeneity were explored were performed. The variables included in the subgroup analyses were study type (Cohort v. Case-control v. Cross-sectional), region of origin (America v. Europe v. Australia v. Asian), mean age of participants (≥65 v.<65 years), classification criteria of AMD (WARMGS v. ICGS v. ICGS and WARMGS) and study quality (Good v. Fair). In addition, sensitivity analyses, by sequentially removing one study at a time, were performed to evaluate whether the results could have been affected markedly by a single study. To assess for publication biases, the Begg’s test, the Egger’s test and funnel plots were evaluated^39^^,40^. We used STATA version 11.0 (Stata Corp LP, College Station, TX) for all statistical analyses. Two-sided P values less than 0.05 were considered statistically significant, except for tests of heterogeneity, Egger’s linear regression and Begg’s rank correlation, where a level of 0.10 was used.

## Additional Information

**How to cite this article**: Ma, L. *et al.* The association between statin use and risk of age-related macular degeneration. *Sci. Rep.* **5**, 18280; doi: 10.1038/srep18280 (2015).

## Figures and Tables

**Table 1:** Characteristics of studies included in this meta-analysis of statins and AMD.

Source	Study type	Country	Sex (%male)	Mean age (years)	AMD type	Diagnosis method	Classification criteria	Adjustment
Klein *et al.*, 2014								
BMES	Cohort	Australia	42.8	63.8	Early and late AMD	Fundus photography	WARMGS	Age, sex, BMI, smoking, diabetes, and hypertension
RS	Cohort	Netherlands	56.3	65.8	Early and late AMD	Fundus photography	WARMGS	Age, sex, BMI, smoking, diabetes, and hypertension
Gre’goire *et al.*, 2014	Cohort	France	38.1	80.2	Early and late AMD	Fundus photography	ICGS	Age, gender, educational level, smoking, BMI, hypertension, HDL, LDL, triglycerides, cardiovascular disease, diabetes, ApoE2, ApoE4, CFH Y402H, ARMS2 A69S, LIPC(rs10468017), LIPC(rs493258) LPL, ABCA1 and CETP polymorphisms
Barbosa *et al.*, 2014	Cross-sectional	US	47.8	68.0	Early and late AMD	Fundus photography	WARMGS	Age, gender, other demographic characteristics, health-related behaviors, comorbidities and self-reported general health condition
Fong *et al.*, 2010	Case-control	US	42.7	72.8	Late AMD	Fundus photography	ICGS	Age, gender, and history of myocardial infarction and of stroke
Maguire *et al.*, 2009	Cohort	US	36.6	70.0	Late AMD	Fundus photography	WARMGS	Age, gender, race, smoking, and hypertension
Smeeth *et al.*, 2009	Cohort	UK	NR	72.1	AMD	Fundus photography	WARMGS	Age, sex, propensity score, year of index date, first diagnosis of any of the following post-index date: diabetes, cerebrovascular disease, *et al.*
Cackett *et al.*, 2008	Cross-sectional	Singapore	NR	56.4	Early and late AMD	Fundus photography	WARMGS	Age, gender, smoking status, hypertension, history of myocardial infarction
Etminan *et al.*, 2008	Case-control	Canada	58.0	70.2	AMD	Fundus photography	ICGS	Age, gender, comorbidity, prior history of diabetic medications, myocardial infarction, stroke, schemic heart disease and congestive heart disease
Tan *et al.*, 2007	Cohort	Australia	42.4	64.4	AMD and early AMD	Fundus photography	WARMGS	Age, gender, smoking, job prestige, history of cardiovascular disease (angina, myocardial infarction, or stroke), white cell count, fibrinogen and total high-density lipoprotein cholesterol
Klein *et al.*, 2007	Cohort	US	52.0	52.0	Early AMD	Fundus photography	WARMGS	Age, sex, race, and study site
McGwin *et al.*, 2006	Case-control	US	40.1	78.3	AMD	Fundus photography	WARMGS	Age, race and sex
Smeeth *et al.*, 2005	Case-control	UK	34.0	74.3	AMD	Eye specialists	WARMGS	Consultation rate, smoking, alcohol intake, BMI, atherosclerosis, hyperlipidemia, heart failure, diabetes, hypertension, cardiovascular drug use, fibrate use
McGwin *et al.*, 2003	Case-control	UK	100.0	73.1	AMD	Fundus photography	WARMGS	Diabetes, lipid metabolism disorders, hypertension, ischemic heart disease, cerebrovascular disease, arterial disease
Klein *et al.*, 2003	Cohort	US	NR	64.0	Early and late AMD	Fundus photography	WARMGS	Age and sex

AMD, age-related macular degeneration; BMES, the Blue Mountains Eye Study; BMI, body mass index; HDL, high-density lipoprotein; ICGS, the International Classification and Grading System; LDL, low-density lipoprotein; NR, not reported; RS, the Rotterdam Study; WARMGS, the Wisconsin Age-related Maculopathy Grading System.

**Table 2:** Stratified analysis of the association between statin use and AMD.

Subgroup	N	Pooled RR	95% CI	P	
				Heterogeneity	Meta-regression
Any AMD	14	0.95	0.74, 1.15	<0.001	NA
Early AMD	7	0.83	0.66, 0.99	0.33	
Cohort	5	0.77	0.56, 0.97	0.19	0.36
Cross-sectional	2	0.98	0.71, 1.26	0.74	
Late AMD	8	0.92	0.77, 1.07	0.63	
Cohort	5	0.95	0.68, 1.22	0.30	0.29
Case-control	1	0.90	0.80, 1.00	NA	
Cross-sectional	2	0.83	0.15, 1.50	0.73	
GA	4	1.16	0.77, 1.56	0.27	
CNV	5	0.90	0.80, 0.99	0.86	0.47
Cohort	4	0.82	0.41, 1.23	0.77	
Case-control	1	0.90	0.80, 1.00	NA	
Study type					
Cohort	7	0.98	0.77, 1.19	0.04	0.78
Case-control	5	0.90	0.55, 1.26	<0.001	
Cross-sectional	2	0.95	0.70, 1.19	0.62	
Region					
America	7	1.03	0.84, 1.23	<0.001	0.67
Europe	5	0.90	0.50, 1.30	<0.001	
Age					
≥65 years	10	0.99	0.75, 1.23	<0.001	0.56
<65 years	4	0.75	0.53, 0.98	0.35	
Classification criteria					
WAMGS	11	0.88	0.74, 1.15	<0.001	0.31
ICGS	3	1.09	0.82, 1.37	<0.001	

AMD, age-related macular degeneration; CNV, choroidal neovascularization; GA, geographic atrophy; ICGS, the International Classification and Grading System; N, number of studies; NA, not applicable because only one study; CI, confidence intervals; RR, pooled relative risk; WARMGS, the Wisconsin Age-related Maculopathy Grading System.

## References

[b1] Lim L. S. *et al.* Age-related macular degeneration. Lancet 379, 1728–1738 (2012). 22559899 10.1016/S0140-6736(12)60282-7

[b2] Seddon J. M., Reynolds R., Yu Y., Daly M. J. & Rosner B. Risk models for progression to advanced age-related macular degeneration using demographic, environmental, genetic, and ocular factors. Ophthalmology 118, 2203–2211 (2011). 21959373 10.1016/j.ophtha.2011.04.029 PMC4097877

[b3] Wong W. L. *et al.* Global prevalence of age-related macular degeneration and disease burden projection for 2020 and 2040: a systematic review and meta-analysis. Lancet Glob Health 2, 106–116 (2014). 10.1016/S2214-109X(13)70145-1 25104651

[b4] Jager R. D., Mieler W. F. & Miller J. W. Age-related macular degeneration. N Engl J Med 358, 2606–2617 (2008). 18550876 10.1056/NEJMra0801537

[b5] van, Lookeren Campagne M., LeCouter J., Yaspan B. L. & Ye W. Mechanisms of age-related macular degeneration and therapeutic opportunities. J Pathol 232, 151–164 (2014). 24105633 10.1002/path.4266

[b6] Erke M. G. *et al.* Cardiovascular risk factors associated with age-related macular degeneration: the Tromsø Study. Acta Ophthalmol 92, 662–669 (2014). 24460653 10.1111/aos.12346

[b7] Wu. J., Uchino M., Sastry S. M. & Schaumberg D. A. Age-related macular degeneration and the incidence of cardiovascular disease: a systematic review and meta-analysis. PLoS One 9, e89600 (2014). 24681973 10.1371/journal.pone.0089600 PMC3969321

[b8] Heart Protection Study Collaborative Group. MRC/BHF Heart Protection Study of cholesterol lowering with simvastatin in 20,536 high-risk individuals: A randomized placebo-controlled trial. Lancet 360, 7–22 (2002). 22115874 10.1016/S0140-6736(11)61125-2 PMC3242163

[b9] Fong D. S. & Contreras R. Recent statin use and 1-year incidence of exudative age-related macular degeneration. Am J Ophthalmol 149, 955–958 (2010). 20346439 10.1016/j.ajo.2009.12.037

[b10] Cackett P. *et al.* Smoking, cardiovascular risk factors, and age-related macular degeneration in Asians: the Singapore Malay Eye Study. Am J Ophthalmol 146, 960–967 (2008). 18723144 10.1016/j.ajo.2008.06.026

[b11] Beri A., Sural N. & Mahajan S. B. Non-atheroprotective effects of statins: a systematic review. Am J Cardiovasc Drugs 9, 361–370 (2009). 19929034 10.2165/11315710-000000000-00000

[b12] Klein R. *et al.* Lipids, lipid genes, and incident age-related macular degeneration: the three continent age-related macular degeneration consortium. Am J Ophthalmol 158, 513–524 (2014). 24879949 10.1016/j.ajo.2014.05.027 PMC4138281

[b13] Cougnard-Grégoire A. *et al.* Elevated high-density lipoprotein cholesterol and age-related macular degeneration: the Alienor study. PLoS One 9, e90973 (2014). 24608419 10.1371/journal.pone.0090973 PMC3946623

[b14] Barbosa D. T. *et al.* Age-related macular degeneration and protective effect of HMG Co-A reductase inhibitors (statins): results from the National Health and Nutrition Examination Survey 2005-2008. Eye 28, 472–480 (2014). 24503725 10.1038/eye.2014.8 PMC3983650

[b15] Maguire M. G. *et al.* Statin use and the incidence of advanced age-related macular degeneration in the Complications of Age-related Macular Degeneration Prevention Trial. Ophthalmology 116, 2381–2385 (2009). 19850347 10.1016/j.ophtha.2009.06.055 PMC2787900

[b16] Smeeth L., Douglas I., Hall A. J., Hubbard R. & Evans S. Effect of statins on a wide range of health outcomes: a cohort study validated by comparison with randomized trials. Br J Clin Pharmacol 67, 99–109 (2009). 19006546 10.1111/j.1365-2125.2008.03308.x PMC2668090

[b17] Etminan M., Brophy J. M. & Maberley D. Use of statins and angiotensin converting enzyme inhibitors (ACE-Is) and the risk of age-related macular degeneration: nested case-control study. Curr Drug Saf 3, 24–26 (2008). 18690977 10.2174/157488608783333952

[b18] Tan J. S., Mitchell P., Rochtchina E. & Wang J. J. Statins and the long-term risk of incident age-related macular degeneration: the Blue Mountains Eye Study. Am J Ophthalmol 143, 685–687 (2007). 17386278 10.1016/j.ajo.2006.11.021

[b19] Klein R. *et al.* Subclinical atherosclerotic cardiovascular disease and early age-related macular degeneration in a multiracial cohort: the Multiethnic Study of Atherosclerosis. Arch Ophthalmol 125, 534–543 (2007). 17420374 10.1001/archopht.125.4.534

[b20] McGwin G. Jr., Modjarrad K., Hall T. A., Xie A. & Owsley C. 3-hydroxy-3-methylglutaryl coenzyme a reductase inhibitors and the presence of age-related macular degeneration in the Cardiovascular Health Study. Arch Ophthalmol 124, 33–37 (2006). 16401782 10.1001/archopht.124.1.33

[b21] Smeeth L., Cook C., Chakravarthy U., Hubbard R. & Fletcher A. E. A case control study of age related macular degeneration and use of statins. Br J Ophthalmol 89, 1171–1175 (2005). 16113375 10.1136/bjo.2004.064477 PMC1772815

[b22] McGwin G. Jr., Owsley C., Curcio C. A. & Crain R. J. The association between statin use and age related maculopathy. Br J Ophthalmol 87, 1121–1125 (2003). 12928279 10.1136/bjo.87.9.1121 PMC1771871

[b23] Klein R., Klein B. E., Tomany S. C., Danforth L. G. & Cruickshanks K. J. Relation of statin use to the 5-year incidence and progression of age-related maculopathy. Arch Ophthalmol 121, 1151–1155 (2003). 12912693 10.1001/archopht.121.8.1151

[b24] Colak E., Majkic-Singh N., Zoric L., Radosavljevic A. & Kosanovic-Jakovic N. The role of CRP and inflammation in the pathogenesis of age-related macular degeneration. Biochem Med 22, 39–48 (2012). 10.11613/bm.2012.005 PMC4062327 22384518

[b25] Ardeljan C. P., Ardeljan D., Abu-Asab M. & Chan C. C. Inflammation and Cell Death in Age-Related Macular Degeneration: An Immunopathological and Ultrastructural Model. J Clin Med 3, 1542–1560 (2014). 25580276 10.3390/jcm3041542 PMC4287551

[b26] Chinwong D. *et al.* Statin therapy in patients with acute coronary syndrome: low-density lipoprotein cholesterol goal attainment and effect of statin potency. Ther Clin Risk Manag 11, 127–136 (2015). 25670902 10.2147/TCRM.S75608 PMC4315463

[b27] Chuo J. Y., Wiens M., Etminan M. & Maberley D. A. Use of lipid-lowering agents for the prevention of age-related macular degeneration: a meta-analysis of observational studies. Ophthalmic Epidemiol 14, 367–374 (2007). 18161610 10.1080/09286580701421684

[b28] Abu-Asab M. S., Salazar J., Tuo J. & Chan C. C. Systems Biology Profiling of AMD on the Basis of Gene Expression. J Ophthalmol 2013, 453934 (2013). 24349763 10.1155/2013/453934 PMC3851728

[b29] Curcio C. A. *et al.* Subretinal drusenoid deposits in non-neovascular age-related macular degeneration: morphology, prevalence, topography, and biogenesis model. Retina 33, 265–276 (2013). 23266879 10.1097/IAE.0b013e31827e25e0 PMC3870202

[b30] Xu Y. *et al.* Statins upregulate cystathionine γ-lyase transcription and H2S generation via activating Akt signaling in macrophage. Pharmacol Res 87, 18–25 (2014). 24951966 10.1016/j.phrs.2014.06.006

[b31] Ridker P. M., Rifai N., Pfeffer M. A., Sacks F. & Braunwald E. Long-term effects of pravastatin on plasma concentration of C-reactive protein. The Cholesterol and Recurrent Events (CARE) Investigators. Circulation 100, 230–235 (1999). 10411845 10.1161/01.cir.100.3.230

[b32] Vine A. K., Stader J., Branham K., Musch D. C. & Swaroop A. Biomarkers of cardiovascular disease as risk factors for age-related macular degeneration. Ophthalmology 112, 2076–2080 (2005). 16225921 10.1016/j.ophtha.2005.07.004

[b33] Seddon J. M., Gensler G., Milton R. C., Klein M. L. & Rifai N. Association between C-reactive protein and age-related macular degeneration. JAMA 291, 704–710 (2004). 14871913 10.1001/jama.291.6.704

[b34] Kim S. Y. *et al.* Morphometric analysis of the macula in eyes with disciform age-related macular degeneration. Retina 22, 471–477 (2002). 12172115 10.1097/00006982-200208000-00012

[b35] Chen J., Connor K. M. & Smith L. E. Overstaying their welcome: defective CX3CR1 microglia eyed in macular degeneration. J Clin Invest 117, 2758–2762 (2007). 17909623 10.1172/JCI33513 PMC1994636

[b36] Moher D. *et al.* Improving the quality of reports of meta-analyses of randomized controlled trials: the QUOROM statement. Quality of Reporting of Meta-analyses. Lancet 354, 1896–1900 (1999). 10584742 10.1016/s0140-6736(99)04149-5

[b37] Stroup D. F. *et al.* Meta-analysis of observational studies in epidemiology: a proposal for reporting. Meta-analysis Of Observational Studies in Epidemiology (MOOSE) group. JAMA 283, 2008–2012 (2000). 10789670 10.1001/jama.283.15.2008

[b38] Begg C. B. & Mazumdar M. Operating characteristics of a rank correlation test for publication bias. Biometrics 50, 1088–1001 (1994). 7786990

[b39] Egger M., Davey Smith, G., Schneider M. & Minder C. Bias in meta-analysis detected by a simple, graphical test. BMJ 315, 629–634 (1997). 9310563 10.1136/bmj.315.7109.629 PMC2127453

